# Blood-Brain Barrier Disruption Induced by Chronic Sleep Loss: Low-Grade Inflammation May Be the Link

**DOI:** 10.1155/2016/4576012

**Published:** 2016-09-21

**Authors:** G. Hurtado-Alvarado, E. Domínguez-Salazar, L. Pavon, J. Velázquez-Moctezuma, B. Gómez-González

**Affiliations:** ^1^Area of Neurosciences, Department of Biology of Reproduction, CBS, Universidad Autónoma Metropolitana-Iztapalapa, Mexico City, Mexico; ^2^Department of Psychoimmunology, National Institute of Psychiatry “Ramón de la Fuente”, Mexico City, Mexico

## Abstract

Sleep is a vital phenomenon related to immunomodulation at the central and peripheral level. Sleep deficient in duration and/or quality is a common problem in the modern society and is considered a risk factor to develop neurodegenerative diseases. Sleep loss in rodents induces blood-brain barrier disruption and the underlying mechanism is still unknown. Several reports indicate that sleep loss induces a systemic low-grade inflammation characterized by the release of several molecules, such as cytokines, chemokines, and acute-phase proteins; all of them may promote changes in cellular components of the blood-brain barrier, particularly on brain endothelial cells. In the present review we discuss the role of inflammatory mediators that increase during sleep loss and their association with general disturbances in peripheral endothelium and epithelium and how those inflammatory mediators may alter the blood-brain barrier. Finally, this manuscript proposes a hypothetical mechanism by which sleep loss may induce blood-brain barrier disruption, emphasizing the regulatory effect of inflammatory molecules on tight junction proteins.

## 1. Introduction

Almost all of our knowledge about the effect of inflammatory events on blood-brain barrier is related to chronic diseases or acute events, in which exacerbated responses to pathogens are present. The role of low-grade inflammation in the generation or exacerbation of neuropathologies is recently explored because several conditions such as obesity and diabetes concur with this inflammatory status during long-term periods and, perhaps, it may be related to systemic and central comorbidities. Most, if not all, pathologies are associated with sleep disturbances. Sleep loss* per se*, including sleep deprivation, sleep restriction, or sleep fragmentation (see Table 1 for a full differentiation between the concepts), generates a pathogen-independent low-grade inflammatory status. Here, we will review (1) the inflammatory mediators that increase during periods of sleep loss and their association with general disturbances in peripheral endothelium and epithelium and (2) how those inflammatory mediators might alter the blood-brain barrier during sleep loss. With the evidence presented in this review, we propose a hypothetical mechanism by which sleep restriction could induce blood-brain barrier disruption, emphasizing the effect of inflammatory molecules on tight junction maintenance.

## 2. Sleep Loss as an Inflammatory Event

Sleep is one of the most widely observed phenomena in mammals and is recognized to play a vital regulatory role in a number of physiological and psychological systems [1, 2]. The paramount role of sleep in the physiology of animal models and humans is evident by the effects of sleep loss. Serious physiological consequences of sleep loss include decreased neurogenesis, cognitive dysfunction (deficits in learning, memory, and decision-making), metabolic alterations, cardiovascular diseases, immune disturbances, and blood-brain barrier disruption [1–8]. Both chronic and acute sleep loss associate with energy balance disturbances [9] and changes in cellular and humoral immunity [10, 11]; however, the direct mechanism by which sleep induces a low-grade inflammatory status is unclear. Experimental research has demonstrated that acute and chronic sleep loss result in impairments in the immune response, characterized by deficits in the cellular component (both in number and in function) and increased levels of proinflammatory mediators, such as tumor necrosis factor-*α* (TFN-*α*), interleukin-1*β* (IL-1*β*), IL-6, IL-17A, and C-reactive protein (CRP) (for details of the cytokine levels related to varying periods of sleep loss see [12]). In addition to immune-derived inflammatory mediators, sleep loss also increases the levels of other inflammatory molecules such as cyclooxygenase-2 (COX-2) [8], nitric oxide synthase (NOS), endothelin-1 (ET-1), vascular endothelial growth factor (VEGF), and insulin-like growth factor-1 (IGF-1) [8, 13].

The major aim of this review is to discuss the role of low-grade inflammation in the blood-brain barrier disruption induced by sleep loss; nevertheless, because endothelial cells form the blood-brain barrier we considered it relevant also to discuss the effect of sleep loss on peripheral endothelial and epithelial cells as early markers of inflammation.

## 3. Peripheral Endothelial and Epithelial Disturbances Induced by Sleep Loss

Endothelial and epithelial cells form protecting barriers in the central nervous system but also in the periphery. Several pathological states are known to target peripheral epithelial and/or endothelial barriers; therefore the knowledge of regulatory mechanisms in those peripheral barriers may contribute to improving the understanding of central barriers. Among the pathologies affecting body barriers, those involving infections and also diabetes, cardiovascular diseases, psoriasis, and cancer are associated with sleep disturbances [14–16]. Here, we present evidence regarding the disrupting effect of sleep loss on peripheral epithelial and endothelial cells.

### 3.1. Humans

When fluid compartmentalization goes awry, homeostasis is altered and the possibility exists of induction of inflammation by microorganism invasion and even of tumor microenvironment induction [14]. In humans sleep restriction increases sympathetic activity and, concomitantly, causes endothelial dysfunction at the venous level [17]; the effect may be mediated via endothelin-1 (ET-1) because ET-1-mediated vasoconstriction is greater in adults with short sleep duration (less than 7 h per night) than in those with normal sleep duration (7–9 h per night) [18]. ET-1 is the most potent vasoconstrictor peptide released by the endothelium. The link between sleep restriction and increased ET-1 activity is not clear, but the role of the inflammatory status induced by sleep loss may partially explain this association. In this way, inflammatory cytokines, insulin, and epinephrine altered during sleep loss have each been shown to increase ET-1 in hypertensive subjects [19]. The cytokines that may increase in sleep-deprived humans (e.g., TNF-*α*, IL-1*β*, and IL-6) raise arterial vascular tone via endothelin receptors [20]. Several reports indicate that sleep loss induces vascular alterations related to inflammatory markers (for a review see [21]). Some studies have tried to clarify the underlying mechanism; for instance, sleep deprivation in humans induced magnesium deficiency [22], which produces arterial constriction, and is a possible cause of myocardial damage [22]. Other barriers are not yet studied in sleep-deprived or sleep-restricted humans, but some studies indicate that sleep deficiency alters skin conductance [23].

### 3.2. Animal Models

Animal models currently used in sleep research include those that model shift work by totally sleep depriving rodents; human sleep deficiency by sleep restricting; and sleep loss-associated with pathologies, such as obstructive apnoea, by promoting sleep fragmentation. Contrary to the human studies, in the case of animal models, several studies have identified negative effects of sleep loss on peripheral endothelia and epithelia. For instance, sleep fragmentation in mice (20 weeks) induces vascular endothelial dysfunction and mild blood pressure increases. Those physiological effects are accompanied by morphological vessel changes characterized by elastic fiber disruption and disorganization, increased recruitment of inflammatory cells to the vessel wall, and increased plasma levels of IL-6 [24]. In rats, total sleep deprivation reduces endothelial-dependent cutaneous vasodilation. This endothelial dysfunction is independent of blood pressure and sympathetic activity but is associated with changes in NOS and COX pathways [25].

The effect of sleep loss on physical barriers such as the intestinal barrier or blood-testis barrier is not reported; however, gut bacteria are present in blood after sleep deprivation [26] and both sleep-deprived and sleep-restricted rats exhibit lower sperm viabilities associated with an increase in endothelial NOS expression [27]. Those data suggest that sleep loss also might alter the physiology of the above-mentioned barriers with the ensuing tissue damage.

## 4. Blood-Brain Barrier Impairment Induced by Sleep Loss

We reported for the first time that sleep restriction induces blood-brain barrier hyperpermeability in rats [7]. We used a procedure consisting of 20-hour sleep deprivation plus 4 hours of sleep opportunity during 10 consecutive days; because a reduction in total sleep time is observed, it is named sleep restriction. In our conditions, rapid eye movement (REM) sleep is fully suppressed and non-REM sleep is 30% reduced since the first day of sleep restriction. In those conditions we showed a widespread breakdown of the blood-brain barrier [7]. We described that brief periods of sleep opportunity (40 to 120 minutes) induced a progressive recovery of blood-brain barrier permeability to Evans blue (>60 000 Da) in the majority of brain regions studied, with exception of the hippocampus and cerebellum [7]. We also observed that in the hippocampus the number of pinocytic vesicles increased threefold. In a subsequent study, mice were subjected to sleep restriction for 6 days in a rotatory bar for 12 hours per day. Sleep restriction by this method induced REM sleep loss in the first 3 days with partial REM sleep recovery afterwards; at the end of the 6th day of sleep restriction, there was 13.3% increase of wakefulness, 10.2% reduction of non-REM sleep, and 2.1% reduction of REM sleep [8]. Under these conditions, increased blood-brain barrier permeability to sodium fluorescein, a low molecular-weight tracer, was observed; sleep recovery by 24 hours fully reverted the effect. In the same way, sleep restriction decreased the mRNA levels of the tight junction proteins claudin-5, zonula occludens-2 (ZO-2), and occludin [8]. In the first study [7] a yoked control was included to avoid any potential confounding effects of stress on blood-brain barrier permeability; rats were placed on large platforms during the same period of time as sleep-restricted subjects and despite being in the same stressful conditions as the sleep-restricted subjects they have a fully functional blood-brain barrier [7]. The second study did not include a yoked control, a newly developed sleep deprivation method was used that involves a rotating bar at the bottom of the house-cage with random changes of direction; this method may certainly be stressful to the rodents due to the presence of forced exercise; however, our recent results replicate their findings (Hurtado-Alvarado et al. personal communication). Therefore, the evidence of changes in the blood-brain barrier integrity induced by sleep loss is substantial and inflammatory molecules appear to play a key role in the mechanism subjacent to this phenomenon.

## 5. Role of Inflammatory Mediators Released during Sleep Loss in Blood-Brain Barrier Physiology

The increase in the levels of inflammatory mediators during chronic sleep loss may be related to blood-brain barrier disruption because several previous reports show that* per se* those inflammatory molecules affect the integrity of the blood-brain barrier (see Table 2 for a summary).

### 5.1. Proinflammatory Cytokines Involved in Sleep and Blood-Brain Barrier Modulation

#### 5.1.1. Tumor Necrosis Factor-*α*

Tumor necrosis factor-*α* (TNF-*α*) is a protein synthesized mainly by monocytes and macrophages that plays an essential role in the initial activation of the immune system. In the central nervous system TNF-*α* is a multipotent cytokine produced by neurons, glia, and microvascular endothelial cells that is implicated in several physiological events, such as memory consolidation and sleep regulation. TNF-*α* is also a potent regulator of blood-brain barrier permeability. The role of TNF-*α* as an inductor of blood-brain barrier disruption includes its overexpression in microglia, astrocytes, and microvascular endothelial cells [28].

Several reports indicate that sleep loss increases the plasma and brain levels of TNF-*α* [29–33], the mRNA expression of TNF-*α* in the brain [33, 34], the spontaneous production of TNF-*α* in lymphocytes [35], and the mRNA expression of TNF-*α* in peritoneal and epididymal adipose tissue [36, 37]. Despite the fact that the changes in TNF-*α* induced by sleep loss are 2 to 5 times higher compared to rats sleeping* ad libitum*, the levels are below those reported in the case of infectious diseases; however, the chronic exposure to this inflammatory mediator may underlie the sleep-induced blood-brain barrier dysfunction.

The effect of TNF-*α* in endothelial cells is well studied.* In vivo* and* in vitro* studies report an increase in the permeability of microvascular endothelial cells after the administration of TNF-*α* in both animal models and human cell lines [38–41]. Nonetheless, the TNF-*α* levels used in those studies are 100,000 times higher compared to concentrations reported under sleep loss conditions. The lower dose of TNF-*α* used in* in vitro* studies (1 ng/mL) results in a transendothelial electric resistance (TEER) reduction at 60 minutes after treatment with TEER recovery at 210 minutes after administration, which is similar to the results observed using higher doses of TNF-*α* (50, 100 ng/mL), suggesting that the effect mediated by TNF-*α* receptors is saturable [42].

While we can infer that peripheral changes mediate the main effect of TNF-*α* on blood-brain barrier, we must not ignore the fact that TNF-*α* levels also increase in the brain. In this way, it is known that after the administration of TNF-*α* (250 ng) in the lateral ventricle an increase in the transport from cerebrospinal fluid (CSF) to blood of ^125^I-human serum albumin is observed in rats, which demonstrates that TNF-*α* promotes the clearance of macromolecules from the CSF to the venous blood [43]. Taking into consideration that the restorative function of non-REM sleep may be a consequence of the enhanced removal of waste products accumulated in the awaking brain via the glymphatic system [44], the TNF-*α* increase during sleep loss may contribute to the clearance of toxins by efflux of potentially neurotoxic waste products via the blood-brain barrier. Interestingly, in the brain, sleep restriction increases the mRNA expression of TNF-*α* in a region-dependent manner in the mouse [45], suggesting that if TNF-*α* regulates the microvascular brain endothelial cells from inside the brain, it may do it in specific areas, such as the somatosensory and frontal cortices, which indicates that blood-brain barrier regulation by inflammatory molecules is heterogeneous (a finding reported by us in the case of blood-brain barrier changes induced by sleep loss and recovery; see [7]).

Another example of TNF-*α* role in blood-brain barrier regulation during peripheral inflammation occurs after the induction of acute pancreatitis in rats, where an increase in TNF-*α* levels is observed as early as 6 hours after pancreatitis induction and at the same time increases the blood-brain barrier permeability to sodium fluorescein (365 Da) in the hippocampus and cerebellum as well as to Evans blue in the hippocampus, basal nuclei, and cerebellum. In the case of the low molecular-weight tracer the normal blood-brain barrier permeability reestablishes at 24 hours after induction, while, for Evans blue, reestablishment occurs 48 hours after induction [46]. We also observed region-dependent effects of sleep loss and recovery on blood-brain barrier integrity; for instance, in the cerebellum the hyperpermeability remained even after sleep opportunity periods of 40–120 minutes; meanwhile the cortex recovered the normal blood-brain barrier permeability at the same time points [7]. Therefore, the cerebellum could be considered as a highly susceptible region to inflammatory mediators such as TNF-*α* [47] in comparison with other brain regions (e.g., the hippocampus and cortex). The differential distribution of TNF-*α* receptors in the brain may explain why TNF-*α* regulates blood-brain barrier function in a region-dependent manner; however, is it also possible that other molecules may have synergic effects with TNF-*α* to regulate blood-brain barrier physiology.

#### 5.1.2. Interleukin-1 Beta

IL-1*β* is the prototypical signal molecule for neuroimmune communication. Classically, phagocytic cells in response to inflammatory stimuli release IL-1*β*; in the brain IL-1*β* activates the regions involved in the generation of hyperthermia [48]. Similar to the effect of TNF-*α*, IL-1*β* administration promotes sleep in mammals [1] and sleep deprivation has been shown to increase serum IL-1*β* levels both in humans and in animal models [3, 4, 29, 49]. In addition, sleep loss induces IL-1*β* gene expression in the brain [34, 45], cardiac muscle, and adipose tissue [36] and on phytohaemagglutinin (PHA) activated peripheral blood mononuclear cells (PBMC) [50]. In the case of the brain, several reports indicate that the expression of the IL-1 receptor-1 (IL-1R1) in endothelial cells is high in the preoptic area, subfornical organ, and supraoptic hypothalamus, while a lesser expression is found in the paraventricular hypothalamus, cerebral cortex, nucleus of the solitary tract, ventrolateral medulla, trigeminal and hypoglossal motor nuclei, and the area postrema [51–53].

In* in vitro* models of blood-brain barrier, IL-1*β* (in doses of 5, 100, and 1000 ng/mL) decreases the TEER similar to the levels observed after TNF-*α* administration [42, 54]. IL-1*β* also promotes the release of IL-6 and prostaglandin E (PGE_2_) in rat brain endothelial cells [55]. Likely,* in vivo* studies have shown that IL-1 induces sickness behaviour mediated by endothelial IL-1R1 activation in rats [56]; the probable mechanism may be the induction of COX-2 in brain endothelial cells after IL-1R1 activation with the concomitant increase in the synthesis of PGE_2_ [57].

IL-1*β* may have a key role in blood-brain barrier dysfunction during sleep loss because it has been reported that sleep loss increases IL-1*β* gene expression in the cerebral cortex, hippocampus, and basal forebrain [45]. In addition, IL-1*β* released from activated microglia increases blood-brain barrier permeability; this effect may depend on the suppression of astrocyte-derived signals that maintain blood-brain barrier integrity (e.g., sonic hedgehog, SHH) [58]. IL-1*β* action on blood-brain barrier may induce the expression of other inflammatory mediators produced by microglia and astroglia. For instance, the lack of IL-1R1 specifically in endothelial cells precluded the brain increase of IL-1*β*, TNF-*α*, and IL-6 in stressed rats despite the presence of reactive microglia [59, 60], which places IL-1*β* and its receptor on endothelial cells as central mediators of brain inflammatory responses. Hence, the role of IL-1*β* in blood-brain barrier could be mainly related to endothelial-glial interactions [61].

#### 5.1.3. Interleukin-6

Sleep onset is associated with an increase in circulating levels of IL-6 [62]; nevertheless, the potential role of IL-6 in sleep regulation is controversial, and it may take a secondary role as compared to its primary role in the acute-phase response [63]. Some studies indicate an increase of IL-6 circulating levels in sleep-deprived subjects [64–66] and also in gene expression in immune cells [35, 50, 67], whereas others report a delay in the sleep-related peak of plasma IL-6 in sleep-restricted subjects [62]. Even some authors report that plasma levels of IL-6 are maintained without change despite sleep loss [30, 68]. Some studies also show that sleep recovery after total sleep deprivation increases plasma levels of IL-6 [69]; however, others found that in immune cells IL-6 levels remain unchanged during sleep recovery [50]. IL-6 is a pleiotropic cytokine key for immune regulation and if secreted during sleep loss and recovery may have neuroprotective effects; indeed, it has been reported that IL-6 appears to be neuroprotective and is involved in endothelial survival after shear stress [70]. However, given the high variability of IL-6 after sleep loss and recovery, the role of IL-6 as a possible modulator of blood-brain barrier during sleep is unclear. It is necessary to elucidate the precise changes in IL-6 levels both centrally and peripherally to clarify the role of IL-6 in blood-brain barrier modulation during sleep.

IL-6 has pyrogenic effects when endogenously released during systemic inflammation; it achieves this function by its binding to IL-6 receptor *α* (IL-6 R*α*) on brain endothelial cells and the subsequent induction of PGE synthesis. However, those effects require high levels of IL-6 (>1 ng/mL). In humans, IL-6 serum levels were less than 100 pg/mL and the normal levels for IL-6 in CSF are around 10 pg/mL, significantly lesser than those measured in several* in vitro* and* in vivo* experiments [70]. For instance, treatment with 50 or 500 ng of IL-6 reduced the infarct volumes and symptoms of neurological deficit in a rat model of cerebral ischemia [71]. In addition, the administration of IL-6 decreased the blood-brain barrier permeability to Evans blue by suppressing the expression of matrix metalloproteinase-9 (MMP-9) [71]. The role of IL-6 as well as TNF-*α* and IL-1*β* may depend on the brain region, for example, the stimulation with lipopolysaccharide (LPS) induces in the brain the expression of the IL-6 receptor (IL-6R) in the cortex and hippocampus but not in the cerebellum [72]. Therefore, considering IL-6 a proinflammatory cytokine it is possible to suggest that its role in blood-brain barrier physiology during sleep loss may be related to the modulation of the expression of other proinflammatory cytokines.

#### 5.1.4. Interleukin-17A

Th17 cells have been identified as a subset of T helper lymphocytes characterized by the production of a number of cytokines including IL-17A, IL-17F, and IL-22. Th17 cells have emerged as a key factor in the pathogenesis of autoimmune disorders. For instance, high expression of IL-17A is associated with autoimmune inflammatory diseases including multiple sclerosis [73], rheumatoid arthritis [74], inflammatory bowel disease [75], and systemic lupus erythematosus [76]. During sleep loss a subtle increase of IL-17A is reported (from 0.5 to 3 ng/mL in rat) [29]. IL-17A high levels were found in plasma even after 24 hours of sleep recovery in sleep-restricted rats [29]. Sleep loss also increases the mRNA and protein expression of IL-17A on PHA activated PBMC in humans [50].

Particularly, the receptor for IL-17A is expressed in epithelial and endothelial cells and promotes the expression of inflammatory mediators such as IL-6 and chemokines [77]. IL-17A induces epithelial and endothelial dysfunction; it decreases the TEER and concomitantly increases tracer permeability; the mechanism is mediated through tight junction disruption [77]. Finally, from* in vitro* experiments it is known that IL-17A increases endothelial cell permeability at 10 or 100 ng/mL doses [78, 79]. These data suggest that IL-17A might be involved in blood-brain barrier disruption during sleep loss.

### 5.2. Other Inflammatory Molecules Altered during Sleep Loss and Their Role in Blood-Brain Barrier Regulation

#### 5.2.1. C-Reactive Protein

C-reactive protein (CRP) is the major acute-phase protein involved in the resistance to microbes and autoimmune diseases and is an important risk marker of cardiovascular and cerebrovascular disorders. The plasma levels of CRP increase faster and at higher magnitude than other acute-phase proteins [80]. Sleep loss increases the circulating levels of CRP (0.5 *μ*g/mL), which is associated with increased risk of cardiovascular disease and stroke [4, 8, 50, 67, 81, 81–83].

The synthesis of CRP in the liver is controlled by proinflammatory cytokines, including TNF-*α*, IL-1*β*, IL-6, and IL-17A [82, 84]. CRP (10–20 *μ*g/mL) induces blood-brain barrier disruption [85] because brain endothelial cells express high levels of CRP receptors (CD16 and CD32) and also because brain endothelial cells express high levels of the p22phox subunit of the NAD(P)H-oxidase. The high expression of both exacerbates the generation of reactive oxygen species (ROS) with the resultant oxidation of tight junction proteins [86].

#### 5.2.2. Intercellular Adhesion Molecule-1 (ICAM-1)

The expression of ICAM-1 in endothelial cells is pivotal in supporting lymphocyte migration across the vascular endothelium [87]. ICAM-1 associates with an endothelial cytoskeleton fraction, suggesting that ICAM-1 redistribution is an early event in the signalling cascade during inflammatory events, particularly in lymphocyte transmigration [87]. The expression of endothelial cell adhesion molecules increases in the central nervous system during inflammation secondary to pathogen intracerebral administration (e.g.,* Corynebacterium parvum*). Brain vessels located in the centre of the cellular infiltrate began to express markers of fenestrate endothelium such as the endothelial-specific expression of MECA32 suggesting an altered functional status of the endothelial cell [88]. Abundant ICAM-1 expression has been observed after IL-1 or TNF-*α* stimulation of cultured heart endothelial cells [89].

Elevated levels of ICAM-1 may contribute to cardiovascular disease and are associated with obstructive sleep apnoea (OSA) and obesity, in which sleep deficiency is present [90]. In the same way, it has been shown that patients with diabetes mellitus type 2 and poor sleep present higher morbidity of cardiovascular diseases than diabetes mellitus patients sleeping normally; those patients also present higher plasma levels of ICAM-1 [91]. ICAM-1 higher serum levels were also found during the sleep recovery period after 40 hours of total sleep deprivation in healthy men [69]. Therefore it seems that the mediator between poor sleep (with bad quality and poor sleep recovery) and higher risk for cardiovascular diseases is ICAM-1.

#### 5.2.3. Vascular Endothelial Growth Factor

Inflammation is characterized by upregulation of vascular endothelial growth factor (VEGF). In* in vivo *experiments, increases in VEGF during neuroinflammation (e.g., in experimental autoimmune encephalomyelitis (EAE)) are accompanied with increased blood-brain barrier permeability and decreased expression of tight junction proteins (e.g., claudin-5 and occludin). Likely, VEGF administration to human brain endothelial cells increases permeability of the monolayer and downregulates claudin-5 and occludin, but not junctional adhesion molecule-1 (JAM-1), cingulin, peripheral plasma membrane protein (CASK), or ZO-1 [92].

Given the role of VEGF in regulating blood-brain barrier during neuroinflammation, it may participate in generating the vascular changes associated with sleep loss. Indeed, it has been shown that VEGF is overexpressed in OSA patients and it is generally considered that VEGF increases are associated with hypoxia events [93]. However, OSA patients also have severe sleep fragmentation; therefore, in addition to chronic intermittent hypoxia, VEGF changes may be related to sleep loss [94]. In fact, in a study with major depressive disorder patients, sleep deprivation increased VEGF plasma levels [95].

#### 5.2.4. Insulin-Like Growth Factor-1

Sleep deprivation decreases IGF-1 levels in rats and humans and one night of sleep recovery is sufficient to restore its basal levels [96]. The neuroprotective effects of IGF-1 are unclear but it is known that IGF-1 receptors are present in brain endothelial cells, microglia, and astroglia and even in neurons [97]. Indeed, it has been suggested that IGF-1 may promote neuroprotection by acting on the blood-brain barrier; in an experimental model of ischemic stroke IGF-1 reduced the inflammatory infiltrate in the brain [97]. In an* in vitro *experiment with brain endothelial cells IGF-1 reverted the hyperpermeability to bovine serum albumin induced by oxygen-glucose deprivation (an* in vitro* model of ischemic stroke) [97].

Changes on inflammatory molecules during sleep loss are well described but we do not know what the source of those alterations is. In this way the role of microbiota could appear a good candidate to induce the low-grade proinflammatory status during sleep loss.

## 6. A Brief View of the Microbiota and Barriers Dysfunction as a Possible Source of Inflammatory Mediators in Sleep-Deficient Subjects

The source of inflammatory mediators during sleep loss remains unclear; however, microbiota may play a key role in this event. In other conditions that exhibit low-grade systemic inflammation, such as chronic depression, obesity, and diabetes, evidence from murine models initially suggested a role for the gut microbiota in the generation of low-grade inflammation, with the consequent increased risk of endothelial and epithelial dysfunction [98, 99]. For instance, changes in gut microbiota composition increase intestinal permeability [100]. In the same way, during sleep deprivation gut microbiota has been detected in blood, suggesting the induction of systemic inflammation and deficits in gut epithelial permeability [26]. In addition, preclinical evidence from germ-free mice suggests that the microbiota can also modulate the blood-brain barrier; exposure of germ-free adult mice to the faecal microbiota from pathogen-free donors decreased the blood-brain barrier permeability and increased the expression of tight junction proteins in brain endothelial cells [101], therefore strengthening the hypothesis that the blood-brain barrier may also be sensible to changes in the gut microbiota composition [100]. The candidate pathways to induce barriers dysfunction under altered gut microbiota composition include serotonin, cytokines, toll-like receptor activation, and short chain fatty acids [100]. Moreover, the inflammatory response subsequent to microbiota-induced barriers disruption may underlie the sleep loss-related cognitive deficits and the exacerbation of neurological disorders such as depression [100].

These data might support the theory of a coevolution between sleep and blood-brain barrier proposed by Korth in 1995 [102]. Because the brain and blood-brain barrier react sensitively to the exposure to bacterial cell wall constituents and sleep is regulated by gut microbiota products, Korth proposed that low amounts of bacterial cell wall constituents that induce sleep under sleep loss conditions, by themselves or by cytokine production, increase the blood-brain barrier permeability ensuing their passage into the brain [102].

## 7. Molecular Mechanisms by Which Inflammatory Mediators Might Induce Blood-Brain Barrier Disruption during Sleep Loss

**Figure 1:**
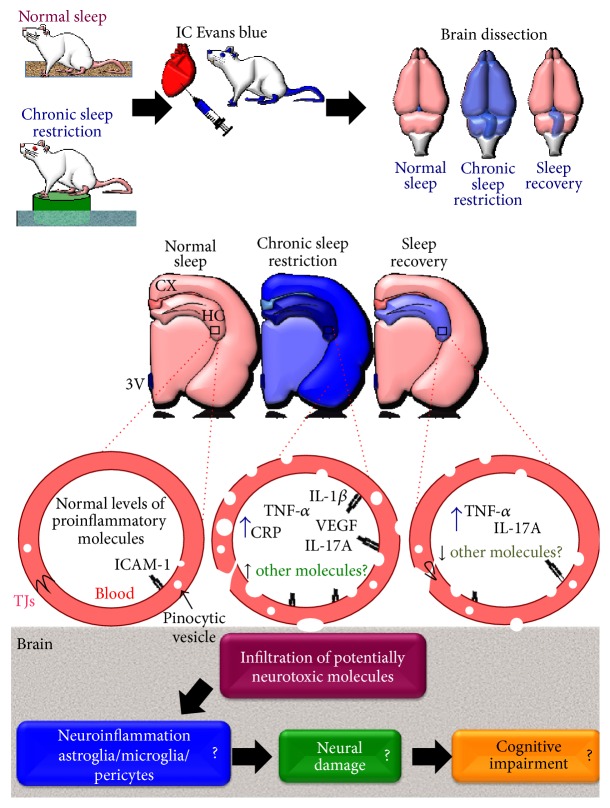
Potential inflammatory mediators participating in the regulation of blood-brain barrier permeability during sleep loss. The figure shows the platform method to induce sleep loss in the rat. Chronic sleep restriction increases blood-brain barrier permeability to circulating molecules (e.g., Evans blue) and sleep recovery promotes restoration of normal blood-brain barrier permeability. Inflammatory mediators with barrier regulation properties, such as tumor necrosis factor-*α* (TNF-*α*), vascular endothelial growth factor (VEGF), interleukin-1*β* (IL-1*β*), and IL-17A, are released during sleep loss conditions and some of them return to basal levels during sleep recovery; others, like IL-17A and TNF-*α*, are maintained at high levels despite sleep recovery. The barrier changes induced by inflammatory mediators may lead to neuroinflammation and potentially may underlie the cognitive impairments induced by sleep loss.

Cytokines and other inflammatory mediators induce blood-brain barrier disruption through mechanisms involving signalling pathways that converge in the disorganization of tight junctions (Figure 1). For instance, it has been reported that proinflammatory cytokines, including TNF-*α* and IL-1*β*, decreased ZO-1 expression and ZO-1-occludin coassociation, concomitant to increased ZO-1 phosphorylation in tyrosine and threonine residues [103]. Those effects are presumably mediated by ROS [103]. ZO-1 phosphorylation in tyrosine residues is also observed after VEGF administration [104]. In this way, VEGF-A also promotes disruption of blood-brain barrier by downregulating the expression of claudin-5 and occludin [92]. Low cytokine concentrations (>1 ng/mL) led to activation of effector caspases via c-Jun N-terminal kinases (JNK) and protein kinase C (PKC) signalling pathways, increased paracellular flux, and redistribution of ZO-1 and VE-cadherin but failed to induce apoptosis [105]. In addition to caspase-3, TNF-*α* activates the production of MMP-9 [106], which is also associated with high levels of IL-1*β* in brain parenchyma [107].

TNF-*α* activates the NF*κ*B signalling pathway, leading to increased PGE levels via COX-2 [108]. COX-2 plays a crucial role in the inflammatory response of the blood-brain barrier (for review see [109]); particularly COX-2 derived PGE_2_ increases blood-brain barrier permeability [110]. Other cytokines, such as IL-1, use other signalling pathways that finally converge in COX-2 induction; particularly, the IL-1 receptor-1 (IL-1R1) signals via the p38 mitogen-activated protein kinase (MAPK) and the c-Jun pathway to induce COX-2 synthesis, whereas activation of the IL-6 receptor leads to COX-2 expression through activation of signal transducer and activator of transcription-3 (STAT-3) [111]. The activation of NF*κ*B by TNF-*α* and IL-1*β* is also correlated with COX-2 expression in microvascular endothelial cells. Indeed, both I*κ*B*α* and COX-2 are expressed within the same endothelial cells, suggesting a potential interaction between the transcription factor and COX-2 expression in the cerebral endothelium of animals with systemic inflammation [112].

TNF-*α* and IL-1*β* promote the release of CRP. The putative mechanism by which CRP increases blood-brain barrier permeability is by its action on CD16/CD32 receptors present in the cell membrane of brain endothelial cells [85]. This association activates the Myosin Light Chain (MLC) phosphorylation by MLC-kinase (MLCK) and the activation of p38-MAPK, with the subsequent formation of actin stress fibers [85]. Brain endothelial cells express the p22phox subunit located in the cell membrane; this enzyme uses NADH or NADPH as the electron donor for the single electron reduction of oxygen to produce ROS during CRP stimulation [86]. The assembly of active NADPH oxidase requires translocation of cytosolic subunits, p47phox, p67phox, and Rac1 (a cytosolic GTPase), to the plasma membrane, where they interact with gp91phox and p22phox and associate with other membrane cofactors to form a functional enzyme complex [113]. In addition, CRP stimulation also disorganizes ZO-1 via MLCK and ROS production [85]. In this way, IL-17A also induces NADPH oxidase- or xanthine oxidase-dependent ROS production and downregulates the expression of occludin by activation of MLCK [79].

The signalling of inflammatory mediators and particularly NADPH oxidase may promote the upregulation of adhesion molecules such as ICAM-1 via JAK/epidermal growth factor receptor (EGFR) signalling [113] contributing to a possible leukocyte infiltration. Therefore, these changes may be deemed as the mechanisms involved in brain endothelial cell dysfunction during sleep loss.

## 8. Conclusion and Future Directions

**Figure 2:**
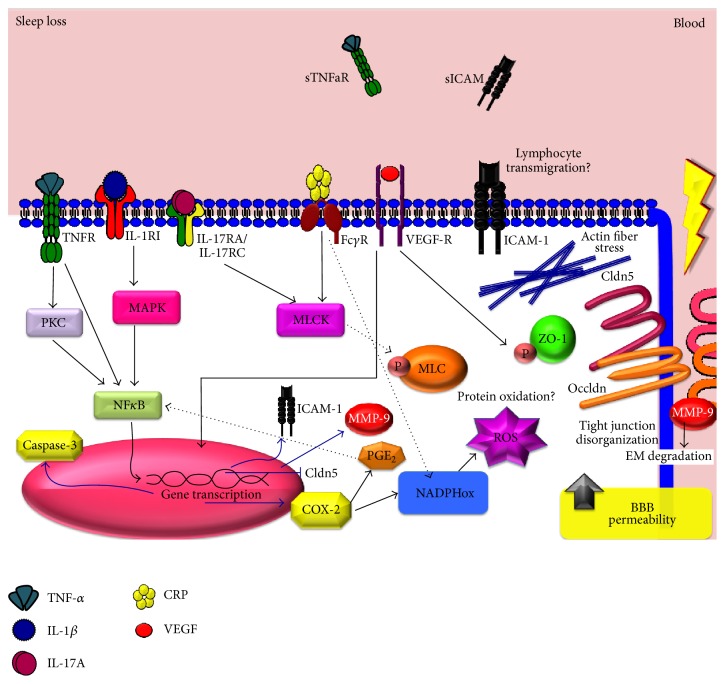
Hypothetical molecular mechanisms mediating sleep loss effect on blood-brain barrier permeability. During sleep loss the increase of soluble inflammatory mediators such as TNF-*α*, IL-1*β*, IL-17A, CRP, and VEGF activates several membrane receptors that converge in cellular pathways hallmark of inflammation, for example, the NF*κ*B pathway. The final outcome involves the phosphorylation of tight junction proteins and the generation of actin fiber stress. But also other pathways are potentially activated, such as the NADPH oxidase pathway, leading to ROS generation and the subsequent lipoxidation and protein oxidation. The activation of transcription factors (eg., NF*κ*B) and their translocation to the nuclei may promote the transcription of inflammatory-related genes (e.g., ICAM-1, prostaglandins, and matrix metalloproteinases (MMP)) as well as death-related genes (e.g., caspase 3) and the repression of genes involved in the maintenance of the barrier properties (e.g., claudin-5). Conjointly, all those pathways could lead to increased blood-brain barrier permeability during chronic sleep loss. Cldn5: claudin-5, COX: cyclooxygenase, CRP: C-reactive protein, Fc*γ* receptor: fragment crystallizable region, ICAM-1: intracellular adhesion molecule-1, IL: interleukin, NADPHox: nicotinamide adenine dinucleotide phosphate oxidase, NF*κ*B: nuclear factor kappa-light-chain-enhancer, MMP: matrix metalloproteinase, MLC: myosin light chain, MLCK: myosin light chain kinase, PGE: prostaglandin, PKC: protein kinase C, sICAM: soluble ICAM, sTNFaR: soluble TNF-*α* receptor, VEGF: vascular endothelial growth factor, TNF: tumor necrosis factor, and ZO: zonula occludens.

We propose that inflammatory mediators increased during chronic sleep loss might promote blood-brain barrier disruption (Figures 1 and 2). For aims of clarity the hypothesis does not explicitly distinguish between REM and non-REM sleep and we know that other molecules altered during sleep loss also should be studied because they may have a potent role in the blood-brain barrier disruption such as adenosine [114] and hormones [115]. In interpreting these data, a number of factors need to be considered. For instance, the cellular components of the blood-brain barrier that promote inflammation in the brain, such as microglia and astroglia, in addition to regulating blood-brain barrier may also be affecting several brain functions during sleep and sleep loss. On the other hand, pericytes have a unique synergistic relationship with brain endothelial cells in the regulation of capillary permeability through secretion of inflammatory mediators including cytokines, chemokines, nitric oxide, and matrix metalloproteinases. Those inflammatory mediators released during sleep restriction may directly induce pericyte detachment from the vessel wall ensuing blood-brain barrier disruption (for review see Hurtado-Alvarado, 2014 [116]).

Summarizing, chronic sleep loss induces systemic low-grade inflammation that may be related to epithelial and endothelial disturbances both at the systemic and at the central level. Particularly, the role of inflammatory mediators in the blood-brain barrier disruption induced by sleep loss might explain the cognitive impairment associated with sleep loss. The systemic and local effect of inflammatory molecules accumulated during chronic sleep loss should be taken into account for the study of general consequences of sleep deficiency including the risk of developing neurologic and neurodegenerative diseases.

## Figures and Tables

**Table 1:** Sleep loss procedures.

	Human procedures	Duration	Animal models	Duration
Sleep deprivation (SD)	(i) Shift working [117] (ii) Voluntary SD [117]	(i) Several days (ii) 12–90 h	(i) Modified multiple platform method (REM SD) [118] (ii) Gentle SD (total SD) [119] (iii) Disk-over-water method (total and selective SD) [120]	(i) 3–96 h (ii) 3–96 h (iii) 3–96 h

Sleep restriction (SR)	(i) Voluntarily SR [117]	(i) 3–5 h	(i) Modified multiple platform method [7] (ii) Rotating bar at the bottom of the house-cage [8]	(i) 20 h of SD plus 4 h of daily sleep recovery (ii) 18 h of SD plus 6 h of daily sleep recovery

Sleep fragmentation (SF)	(i) Obstructive apnoea patients [117] (ii) The elderly [117]	(i) Several days (ii) Several days	(i) Gentle manipulation coupled to EEG recording [119] (ii) Disk-over-water method [120]	(i) 1 to several days (ii) 1 to several days

Sleep deprivation consists of sleep loss without sleep opportunity along a short period; sleep restriction consists of a reduction in total sleep time with short periods of sleep opportunity; and sleep fragmentation consists of multiple awakenings during sleep time.

**Table 2:** Inflammatory mediators released during sleep loss that may potentially regulate blood-brain barrier integrity.

Inflammatory mediator	General changes during sleep loss	General effects on blood-brain barrier
TNF-*α*	↑ circulating levels in human and rodents [29–32] ↑ mRNA expression in mice brain [33]	↑ blood-brain barrier permeability in *in vivo* and *in vitro* models (rodent and human brain endothelial cells) [38–40] ↑ efflux of albumin from brain to blood [43] ↓ ZO-1 expression [103] ↑ MMP-9 protein expression [106]

IL-1*β*	↑ circulating levels in human and rodents [3, 4, 29, 49] ↑ mRNA expression in mice brain [45]	↑ blood-brain barrier permeability in *in vivo* and *in vitro* models (rodent and human brain endothelial cells) [42, 54] ↓ TEER of primary cultures of brain endothelial cells and human brain endothelial cells [42, 54] ↑ production of PGE and COX [57] ↓ ZO-1 expression [103]

IL-6	↑ circulating levels in human after chronic sleep loss [64, 65] ↓ circulating levels in humans [62] ↑ circulating levels during sleep recovery in humans [69] ↑ mRNA expression in human PBMC [50, 67]	↓ TEER in cerebrovascular endothelial cells from rats at higher doses but not at lower doses [42] ↓ blood-brain barrier permeability in ischemic brain in rodents [71]

IL-17A	↑ circulating levels in rodents [29] ↑ mRNA expression in human PBMC [50]	↑ blood-brain barrier permeability in *in vivo* and *in vitro* models (rodent and human brain endothelial cells) [78, 79]

CRP	↑ circulating levels in humans and rodents [4, 8, 50, 67, 81–83]	↑ blood-brain barrier permeability in *in vivo* and *in vitro* models (rodent and human brain endothelial cells) [85] ↑ ROS production in brain endothelial cells [86]

TNF: tumor necrosis factor; IL: interleukin; CRP: C-reactive protein; ZO: zonula occludens; MMP-9: matrix metalloproteinase-9; PBMC: peripheral blood mononuclear cells; ROS: reactive oxygen species; COX; cyclooxygenase; and TEER: transendothelial electric resistance.

## Abbreviations

CSF: Cerebrospinal fluid
COX: Cyclooxygenase
CRP: C-reactive protein
CXCL-1: Chemokine (C-X-C motif) ligand 1
EGFR: Epidermal growth factor receptor
ET-1: Endothelin-1
ICAM-1: Intracellular adhesion molecule-1
IGF-1: Insulin-like growth factor-1
I*κ*B*α*: Nuclear factor of kappa light polypeptide gene enhancer in B-cells inhibitor, alpha
IL: Interleukin
JNK: c-Jun N-terminal kinase
NADPH: Nicotinamide adenine dinucleotide phosphate
NF*κ*B: Nuclear factor kappa-light-chain-enhancer
NOS: Nitric oxide synthase
MMP: Matrix metalloproteinase
MLC: Myosin light chain
MLCK: Myosin light chain kinase
PBMC: Peripheral blood mononuclear cells
PGE: Prostaglandin
PHA: Phytohaemagglutinin
PKC: Protein kinase C
REM: Rapid eye movement
STAT: Signal transducer and activator of transcription
TEER: Transendothelial resistance
TNF: Tumor necrosis factor
VEGF: Vascular endothelial growth factor
ZO: Zonula occludens.
